# Liquefying Flavonoids with Terpenoids through Deep Eutectic Solvent Formation

**DOI:** 10.3390/molecules27092649

**Published:** 2022-04-20

**Authors:** Gabriel Teixeira, Dinis O. Abranches, Liliana P. Silva, Sérgio M. Vilas-Boas, Simão P. Pinho, Ana I. M. C. L. Ferreira, Luís M. N. B. F. Santos, Olga Ferreira, João A. P. Coutinho

**Affiliations:** 1 CICECO-Aveiro Institute of Materials, Department of Chemistry, University of Aveiro, 3810-193 Aveiro, Portugal; gabriel.teixeira@ua.pt (G.T.); jdinis@ua.pt (D.O.A.); lilianapatrocinio@ua.pt (L.P.S.); sergiovboas@ipb.pt (S.M.V.-B.); 2 Mountain Research Center (CIMO), Polytechnic Institute of Bragança, Campus de Santa Apolónia, 5300-253 Bragança, Portugal; spinho@ipb.pt; 3 CIQUP, Institute of Molecular Sciences (IMS)—Departamento de Química e Bioquímica, Faculdade de Ciências da Universidade do Porto, Rua Campo Alegre, 4169-007 Porto, Portugal; ana.ferreira@fc.up.pt (A.I.M.C.L.F.); lbsantos@fc.up.pt (L.M.N.B.F.S.)

**Keywords:** deep eutectic solvents, solid-liquid equilibria, flavonoids, terpenoids, COSMO-RS

## Abstract

The formation of deep eutectic solvents (DES) is tied to negative deviations to ideality caused by the establishment of stronger interactions in the mixture than in the pure DES precursors. This work tested thymol and menthol as hydrogen bond donors when combined with different flavonoids. Negative deviations from ideality were observed upon mixing thymol with either flavone or flavanone, two parent flavonoids that only have hydrogen bond acceptor (HBA) groups, thus forming non-ionic DES (Type V). On the other hand, the menthol systems with the same compounds generally showed positive deviations from ideality. That was also the case with the mixtures containing the more complex hydroxylated flavonoid, hesperetin, which resulted in positive deviations when mixed with either thymol or menthol. COSMO-RS successfully predicted the behavior of the solid-liquid phase diagram of the studied systems, allowing for evaluation of the impact of the different contributions to the intermolecular interactions, and proving to be a good tool for the design of DES.

## 1. Introduction

The search for less toxic or hazardous solvents produced from renewable resources represents an important step toward designing sustainable processes in different areas [1,2,3]. In that perspective, deep eutectic solvents (DES) were proposed as a new class of solvents with the potential to address sustainability and environmental issues [4,5]. DES are tunable solvents that can be designed to become a greener (less-toxic and more sustainable) alternative for many applications [6,7,8]. Another advantage of DES is their preparation, as they can be obtained in a synthesis-free fashion by mixing two or more pure compounds. The eutectic mixture is classified as deep if its melting temperature is lower than that predicted, assuming ideal thermodynamic behavior [9]. Otherwise, if the mixtures behave ideally, they should be classified as eutectic mixtures or eutectic solvents without using the prefix deep [9]. The key to the non-ideality of a DES is the formation of favorable intermolecular interactions between the DES precursors, primarily through intermolecular hydrogen bonds between hydrogen bond donors (HBD) and acceptors (HBA) [9,10,11]. These interactions must be stronger than the interactions existing in the pure compounds. Earlier works in the field relied on ionic compounds, particularly quaternary ammonium salts such as choline chloride, to formulate DES with strong intermolecular interactions [8,12,13,14]. However, strongly interacting DES can also be obtained using non-ionic compounds such as HBA and HBD, which are classified as type V [11,15].

The focus of this work is the design of DES for the extraction of flavonoids from plant matrices. Flavonoids are polyphenols and secondary metabolites widely present in plant cells that can find application as natural preservatives, nutraceuticals, or drugs due to their biological activities (e.g., antimicrobial, antioxidant, anti-inflammatory, and cardioprotective) [16,17,18]. Several eutectic solvents have already been applied in the literature not only as extraction solvents of polyphenols, but also as potential formulation media in cosmetic, pharmaceutical, or food areas. The most common mixtures include choline chloride, polyalcohols, organic acids, amino acids, sugar, or amides [2,17,18,19].

In most works published so far, the design of DES results from extensive laboratory work, usually by performing time-consuming extraction assays to screen endless combinations of HBD and HBA at different stoichiometric ratios and to optimize extraction parameters (e.g., water content, temperature, operation time, solid-liquid ratio, and stirring rate). More fundamental studies are needed to understand the molecular interactions in the liquid phase, between solvents and solutes, and provide a more efficient design of the best DES for a given application. Some success on the *a priori* design of DES has been achieved using the Conductor-like Screening Model for Real Solvents (COSMO-RS) [15,20,21,22]. This statistical thermodynamics-based model uses a quantum chemistry description of molecules (σ-profiles, calculated from σ-surfaces) to predict their chemical potential (and derived properties, namely activity coefficients and excess properties) in liquid mixtures [23,24,25]. These σ-surfaces are obtained from density functional theory (DFT) calculations, where the geometry of each molecule of interest is optimized by embedding it in a continuum solvent through the formation of a cavity, on top of which screened charges (σ) are calculated. As such, it can be used (i) to quickly screen a wide range of precursors to find viable DES-forming pairs that establish strong intermolecular interactions when mixed and (ii) to predict the solid-liquid equilibria (SLE) of a DES, provided their fusion properties are known.

Previous works suggested that type V DES should be formulated using HBA, such as alcohols, ketones or amines, and phenolic compounds acting as HBD [11,15]. Given that flavonoid molecules are rich in HBA sites, phenolic-based HBD could be applied to extract flavonoids from natural matrices through “in situ” DES formation by creating specific interactions with the HBD and/or HBA groups of the solutes. These interactions are particularly strong when the HBA cannot form hydrogen bonds with itself (which may be referred to as lone HBA [15]), as observed in betaine-based [10,26], trioctylphosphine oxide-based (TOPO-based) [20,27,28], and diazabicyclo-based systems [29].

The two most commonly used compounds to prepare type V DES are thymol, which acts as a strong HBD, and menthol, which can act as an HBD or an HBA [22,30,31,32]. It is important to note, though, that the use of these compounds does not guarantee non-ideal behavior, which reinforces the need to assess the solid-liquid phase diagram of novel mixtures [33,34]. For example, Martins et al. [33] reported that phase diagrams for mixtures of thymol with monocarboxylic acids are mostly ideal. The reason behind this behavior is related to the fact that both compounds have similar acidities, and thus no novel stronger interactions are expectable in the mixture.

**Figure 1:**
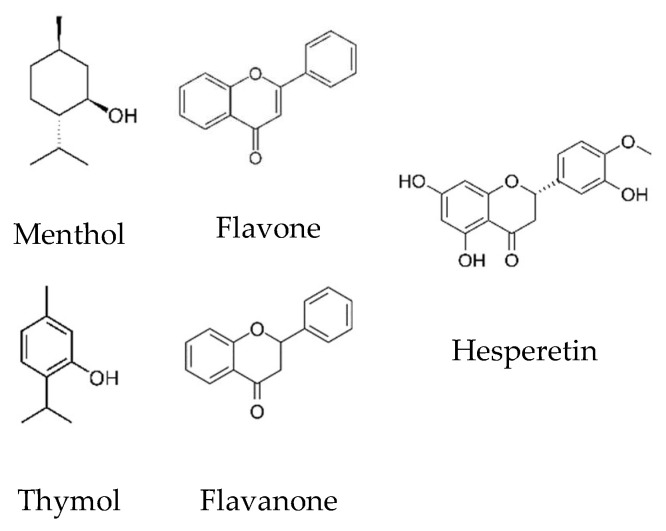
The chemical structure of the compounds studied in this work.

In this work, flavone, flavanone, and (-)-hesperetin ((2S)-3′,5,7-trihydroxy-4′-methoxyflavan-4-one) were selected as representatives of flavonoids present in plants, and their capability of forming DES with menthol or thymol was evaluated. Their structures are presented in Figure 1. Note that flavone and flavanone are examples of lone HBA while hesperetin possesses both HBD and HBA capability. The SLE phase diagrams of these mixtures were experimentally measured using an automatic melting point glass capillary device or by Differential Scanning Calorimetry (DSC). Complementarily, the SLE phase diagrams were predicted using COSMO-RS to provide insights into the molecular interactions in these systems and to evaluate the ability of the model to design DES.

## 2. Results

**Figure 2:**
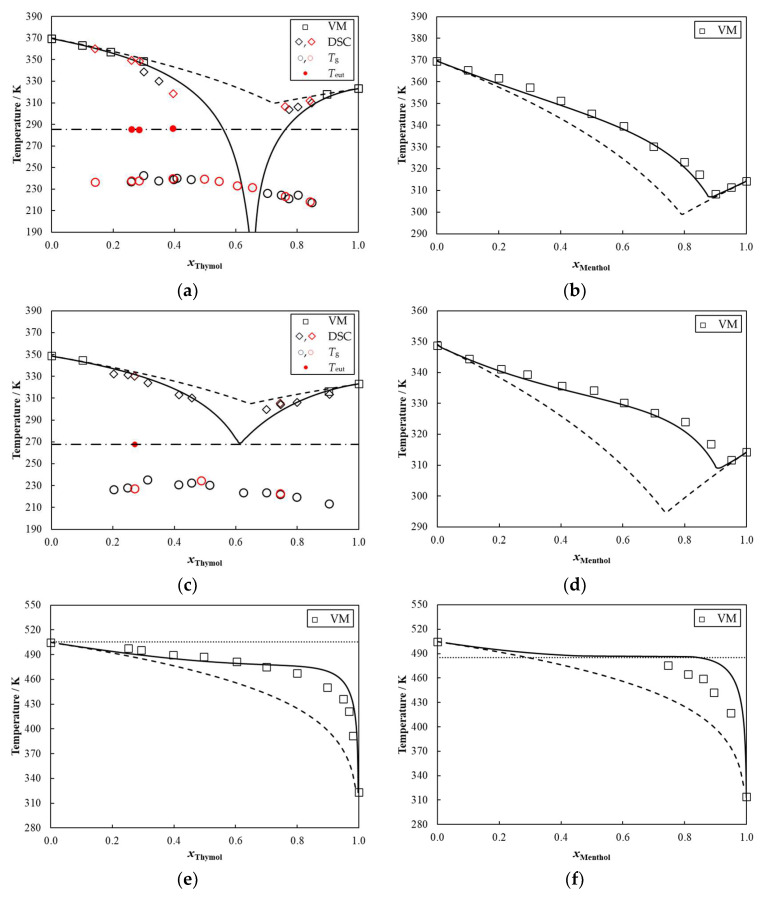
Solid-liquid phase diagrams of (**a**) thymol/flavone, (**b**) menthol/flavone, (**c**) thymol/flavanone, (**d**) menthol/flavanone, (**e**) thymol/hesperetin and (**f**) menthol/hesperetin. Symbols represent experimental melting temperatures, measured using the visual method—VM (□) or DSC (◇), glass transition temperatures (*T_g_*) measured using DSC (○), and eutectic temperatures *T_eut_* (•). The DSC measurements and glass transition temperatures in black were obtained with DSC 1 and the red ones, with DSC 2. Lines represent the COSMO-RS predictions (—), ideal melting curves of the system (— —), eutectic temperature line (— • —), and the terpenoid normal boiling temperature (•••).

The SLE phase diagrams measured in this work are depicted in Figure 2 and reported in Tables S1–S6 (in Supporting Information). Only the visual method was applied for menthol-containing systems as the prepared mixtures were solid at room temperature. It was thus possible to measure the liquidus line of the menthol/flavone and menthol/flavanone diagrams in the whole composition range. Both systems presented a eutectic point at temperatures close to or above 310 K and a menthol mole fraction around 0.9. However, the thymol/flavone and thymol/flavanone systems showed strikingly different behavior. Here, the eutectic point is located below room temperature and, thus, mixtures in the liquid state were formed in the middle range of compositions, with a eutectic point located at a thymol mole fraction slightly above 0.6. For these two mixtures, complementary studies were performed by DSC. During those measurements, glass transitions were observed, and most systems presented their crystallization in the heating step. Furthermore, the DSC curves rarely showed eutectic fusion transitions (solidus line) and some curves presented neither crystallization nor fusion transitions, only the glass transition. This behavior in mixtures with thymol was previously discussed by Alhadid et al. [35] who attributed the glass state formation to inefficient crystal packing and strong intermolecular interactions. Nevertheless, it was possible to obtain the eutectic temperature for the system thymol/flavone (*T*_eut_ = 286 K) and thymol/flavanone (*T*_eut_ = 268 K). Figure S1 shows some examples of the DSC curves obtained for these mixtures.

In the case of menthol/hesperetin mixtures, the liquidus line could not be measured in an extensive composition region since its temperatures approached the normal boiling temperature of menthol (485 K [36]). Therefore, only a few points could be obtained for *x*_menthol_ ≥ 0.75. A similar situation occurred for thymol/hesperetin mixtures when the liquidus line temperatures were close to the normal boiling temperature of thymol (505 K [37]).

In Figure 2, the experimental data are compared to the predictions of the ideal liquid phase model. Very distinct behaviors were observed for the systems containing thymol or menthol and flavone or flavanone. Those compounds mixed with thymol showed negative deviations from ideality in the entire composition range, while the systems with menthol presented positive deviations. For the mixtures containing hesperetin, positive deviations from ideality were consistently obtained. Therefore, only the systems thymol/flavone and thymol/flavanone can be classified as deep eutectic solvents. From an application point of view, the negative non-ideality of flavone and flavanone with thymol could lead to their selective extraction from organic matrices containing all three flavonoids studied, thus reiterating the importance of evaluating the non-ideality of novel DES.

The COSMO-RS predictions of the phase diagrams are also shown in Figure 2. The performance of this model is remarkable, as it can quantitatively predict the SLE phase diagrams of all the systems studied, with the exception of a small composition window in the thymol/flavone system, where the eutectic temperature appears to be underestimated. These results reinforce COSMO-RS as a valuable and fundamental tool to screen precursors for the DES formation, as advocated in previous works [20,21,38]. In addition, it is worth noting that the COSMO-RS model was developed for non-ionic mixtures, which makes it particularly useful and accurate for the formulation of type V DES.

**Figure 3:**
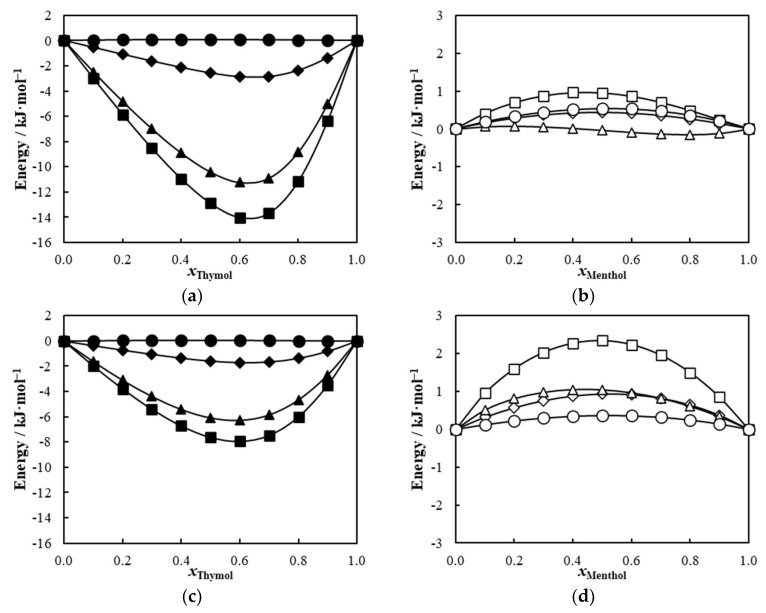

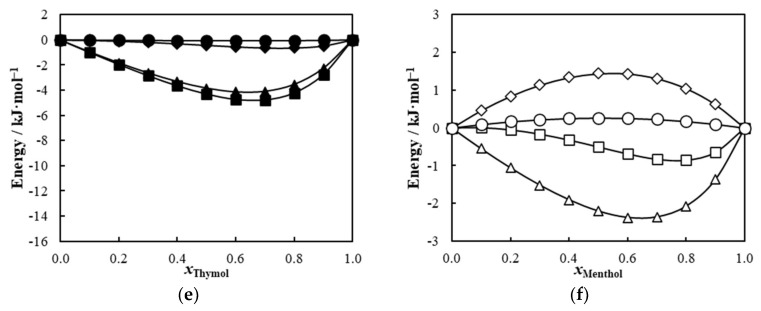
Excess enthalpies predicted by COSMO-RS, at 298.2 K, for the systems thymol/flavone (**a**), menthol/flavone (**b**), thymol/flavanone (**c**), menthol/flavanone (**d**), thymol/hesperetin (**e**), and menthol/hesperetin (**f**). The symbols represent total excess enthalpy (□), misfit contribution (◇), hydrogen bond contribution (△), and van der Waals contribution (○).

In a decade-old review, the overall accuracy of COSMO-RS to estimate transfer free energies or enthalpies of neutral compounds was approximately 1.3 kJ mol^−1^ (quantified as a root mean squared deviation) using COSMOtherm parameterization cited in that work [39]. Nevertheless, inspired by the success of COSMO-RS in predicting the SLE phase diagrams discussed above, and to further understand the dominant intermolecular interactions present in the systems reported in Figure 2, their excess enthalpies were estimated using COSMO-RS and separated into three contributions: hydrogen bonds (HB), misfit (electrostatic interactions that do not include HB), and van der Waals (dispersion forces) (see Figure 3 and Figure S2). The qualitative and quantitative differences between the systems are discussed below, though the model’s estimated accuracy should be kept in mind, particularly in the menthol-containing systems in Figure 3 and the hesperetin systems in Figure S3.

The systems with thymol all present negative excess enthalpies clearly dominated by hydrogen bonding interactions with a negligible impact of the dispersive forces. Contrary to that which is often suggested, COSMO-RS results indicate that π-π interactions do not play any role in these systems. The systems with menthol present a much different behavior with small positive excess enthalpies for the systems with flavone and flavanone, where for the first case, the hydrogen bonding seems to have a negligible contribution while for the latter, it adds to the positive deviations, suggesting that unfavorable hydrogen bonding is taking place in this system. A small negative excess enthalpy is observed for the hesperetin containing systems, dominated by the hydrogen bonding contribution.

The differences found between the thymol and menthol systems with either flavone or flavanone are very interesting. Abranches et al. [11] already discussed how the resonance structures of thymol render the hydrogen of its hydroxyl group a better HBD site and the oxygen a worse HBA site, while menthol has a regular hydroxyl group. Lone HBAs, such as flavone and flavanone, have also been proposed as powerful DES-forming compounds [15]. Thymol mixed with flavone or flavanone fits that criterion resulting in negative deviations from ideality. As shown in Figure 3, the total excess enthalpy of thymol mixtures, calculated at 298.2 K, resulted in minimum negative values of −14 kJ·mol^−1^ for the flavone system and −8 kJ·mol^−1^ for flavanone. These negative values are related to stronger interactions between unlike molecules that are by far ruled by the hydrogen bond contribution (about 80% of the total). In fact, stronger HB interactions can be established in the mixture between the stronger HBD site of thymol and the regular lone HBA sites of flavone/flavanone compared to the existing interactions in the pure compounds. On the other hand, the total excess enthalpies of menthol mixtures are positive (maximum values of +1.0 kJ·mol^−1^ for flavone and +2.4 kJ·mol^−1^ for flavanone, respectively), resulting from the smaller and more balanced contributions of the misfit, van der Waals, and HB interactions. In this case, the HB interaction menthol/flavone has a minimum negative contribution of −0.2 kJ·mol^−1^ while for menthol/flavanone it shows a positive maximum of +1 kJ·mol^−1^ to the total excess enthalpy.

**Figure 4:**
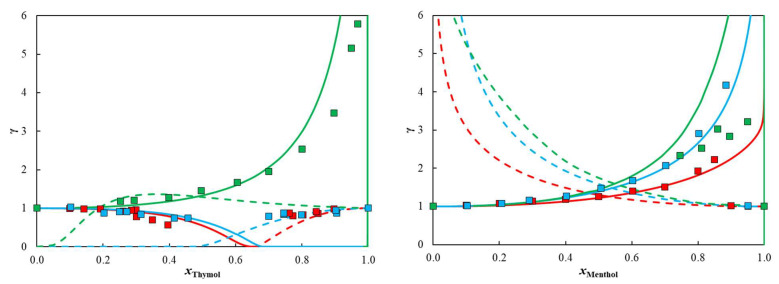
Non-isothermal activity coefficients predicted by COSMO-RS whilst calculating their solid-liquid equilibrium diagram. Symbols represent experimental activity coefficients obtained through Equation (2). Full lines represent flavone (red), flavanone (blue), or hesperetin (green) and the dashed lines represent the second component (thymol or menthol).

From both the activity coefficient data (experimental and predicted by COSMO-RS in Figure 4) and excess enthalpy calculations, the same picture emerges showing that the intermolecular interactions that thymol can establish are stronger than menthol, while the interactions of both with flavone are stronger than with flavanone, suggesting that flavone is a better HBA than flavanone. This is consistent with the sigma-profiles of the flavonoids, predicted by COSMO-RS (see Figure S4), in which both molecules present the highest peaks in the apolar region (−0.01 < σ < 0.01), representing the apolar benzene rings, and one smaller peak in the negatively polar region (σ > 0.01), representing the ether and ketone polar segments. For flavone, this smaller peak is slightly shifted to the right of the diagram, meaning that those groups are more negatively charged than the ones present in flavanone. This difference stems from resonance effects which are stronger in flavone than in flavanone (note how the hybridization of all atoms in flavone is sp2 while flavanone possesses two sp3 carbons, preventing resonance with the second benzene ring).

Moving to the more complex hydroxylated flavonoid, when mixing hesperetin with either thymol or menthol, positive deviations to ideality were obtained, with experimental activity coefficients of hesperetin higher than one (experimental values available in Tables S5 and S6, and values predicted by COSMO-RS in Figure 4). This positive deviation from ideality (and consequent dampening of the melting temperature depression), as will be discussed in detail, is the result of (i) the HBD capability of hesperetin and (ii) its high melting temperature.

As depicted in Figure 1, hesperetin possesses phenolic hydrogen groups similar to thymol. As such, it already establishes strong hydrogen bonding with itself and, as pointed out above, does not lead to strong negative deviations from ideality when mixed with thymol. This effect is quantified by the excess enthalpies predicted for thymol/hesperetin (−5 kJ·mol^−1^) which are much smaller than those for thymol/flavone or thymol/flavanone. However, while the excess enthalpies of thymol/hesperetin (−5 kJ·mol^−1^) and menthol/hesperetin (−1 kJ·mol^−1^) are small and would imply a near-ideal system, Figure 2 shows strong positive deviations from ideality. As discussed below, this discrepancy can be rationalized in terms of the temperature dependence of hydrogen bonding.

The melting temperature of hesperetin (505 K) is much higher than those of flavone or flavanone (370 K and 349 K, respectively). As such, SLE is shifted to higher temperatures, where hydrogen bonding becomes weaker. For comparison, the excess enthalpy predicted by COSMO-RS at 440 K (close to the lowest temperature of SLE measured experimentally) is shown in Figure S3. At this temperature, the total excess enthalpy of the thymol/hesperetin mixtures is positive (maximum of +1 kJ·mol^−1^) and the HB contribution is also slightly positive (maximum + 0.08 kJ·mol^−1^). This indicates that the interactions in the pure compounds are stronger than in the mixture which is in agreement with the hesperetin activity coefficients in thymol. This hydrogen bond weakening due to temperature-induced effects has already been reported and interpreted before for the prototypical type V DES thymol/menthol [40].

In the case of hesperetin (and other hydroxylated flavonoids), as it has a phenolic compound with three hydroxyl groups, a good approach would be its combination with compounds containing lone HBA sites. When searching for cocrystals of hesperetin [41], Chadha et al. (2017) obtained eutectic systems of hesperetin with one of four compounds (gallic acid, theophylline, adenine, and theobromine). At least the latter three contain lone HBA sites. The uncertainty associated with the melting enthalpies of those compounds is high, as all four compounds have melting points even higher than hesperetin, which hinders the confident prediction of the ideal solubility curves (see Figure S5). Nevertheless, the eutectic temperatures vary between 465 K for the theophylline-containing system and 494 K for adenine, and the activity coefficients could be calculated (see Figure S6). It shows positive deviations to ideality for hesperetin and negative deviations for the second compounds. These results and the ones obtained in this work suggest further studies with compounds containing lone HBA sites, preferably with a lower melting point.

## 3. Materials and Methods

### 3.1. Chemicals

Information regarding the CAS number, supplier, and purity of the compounds studied in this work can be found in Table 1. Their melting properties—temperature (*T*_m_) and enthalpy (Δ_m_*H*)—are also reported. The water content of all compounds was evaluated via Karl-Fischer titration. Thymol was dried and purified under vacuum (<10 Pa) at just above its melting point.

### 3.2. Mixture Preparation

For each system, mixtures were prepared to cover the entire composition range. For the visual method measurements, the mass of each component was weighed using an analytical balance model ALS 220-4N from Kern (Balingen, Germany) with an accuracy of ±0.002 g. For the DSC measurements, the mixtures were handled inside a dry-nitrogen glovebox at room temperature, while the mass of each component was weighed using an analytical balance model AG245 from Mettler Toledo (Greifensee, Switzerland) with a mass resolution of ±0.01 mg. To ensure the homogeneity of the mixtures, the flasks containing the compounds were heated, with continuous stirring, until fusion. Then, the mixtures were allowed to cool down to room temperature for at least 24 h until recrystallization. The solid systems were then powdered in a mortar and pestle until a fine powder, capable of being inserted in a glass capillary, was obtained. Alternatively, if at least one of the compounds degraded with heat, the system was directly crushed, skipping the heating process [10,34,44].

### 3.3. Solid-Liquid Equilibria Measurement

The solid-liquid equilibria (SLE) phase diagrams were experimentally measured using two techniques. For the mixtures in the solid state at room temperature, it was possible to apply a visual method, using an automatic glass capillary device model M-565 from Büchi (Flawil, Switzerland) with temperature resolution of 0.1 K. At least three independent measurements were carried out, applying a heating rate of 0.2 or 0.1 K·min^−1^.

For the systems only partly in the solid state at room temperature, DSC was applied. A mixture sample (between 5 and 25 mg) was transferred to an aluminum crucible and sealed. The measurements were made using two sets of equipment, DSC 204 F1 Phoenix^®^ from Netzsch (Selb, Germany) (DSC 1) and Pyris Diamond DSC from Perkin Elmer (Villepinte, France) (DSC 2). Both pieces of equipment were calibrated (temperature and power scales) with several standards as described in previous works [45]. Firstly, samples were cooled to 217.15 K at 10 K·min^−1^ and then heated at 5 K·min^−1^ until 20 K above the glass transition temperature. Since most systems presented cold crystallization phenomena, the next procedure was based on at least two heating (at 1 K·min^−1^) and cooling (at 10 K·min^−1^) cycles to ensure completion of the crystallization transition, which was not always evident. Then, the last heating step at 5 K·min^−1^ was performed for thermal analysis until the complete melting. Thermal transitions were taken as the peak temperature. 

### 3.4. Thermodynamic Framework

An SLE diagram relates the phase transitions of a mixture of two or more compounds with its molar composition. The term eutectic is defined as the isothermal reversible reaction in which one liquid phase is transformed into two (or more) different solid phases during the cooling of a system [46]. Assuming a pure solid phase and neglecting the influence of temperature on the heat capacities and any phase transitions other than the fusion, the melting curves can be described by Equation (1),

(1) $$\ln\left(x_{i}\gamma_{i}\right)=\frac{\Delta_{m}H}{R}\left(\frac{1}{T_{m}}-\frac{1}{T}\right)+\frac{\Delta_{m}C_{p}}{R}\left(\frac{T_{m}}{T}-\ln\frac{T_{m}}{T}-1\right),$$

where $\gamma_{i}$ is the activity coefficient of compound *i* at mole fraction $x_{i}$ in the liquid phase, $\Delta_{m}H$ and $T_{m}$ are the melting enthalpy and temperature of pure compound *i*, R is the universal gas constant, T is the absolute temperature, and $\Delta_{m}C_{p}$ is the difference between the molar heat capacity of compound *i* in the liquid and solid phases. Although the heat capacity difference is often unavailable, the second term of Equation (1) can be neglected [9,47] if the difference between T and $T_{m}$ is lower than 100 K, and, in that case, simplified into Equation (2),

(2) $$\ln\left(x_{i}\gamma_{i}\right)=\frac{\Delta_{m}H}{R}\left(\frac{1}{T_{m}}-\frac{1}{T}\right)$$

Equation (2) was used in this work, in combination with the melting properties listed in Table 1, to calculate the solid-liquid phase diagram of the flavonoid-based systems studied and the ideal melting curves taking $\gamma_{i}\;$= 1.

### 3.5. The COSMO-RS Model

COSMO-RS was used in this work to predict the SLE phase diagrams of the flavonoid-based systems studied. To do so, Equation (2) is solved in an iterative, self-consistent manner, by varying the temperature and using COSMO-RS to compute the activity coefficient ($\gamma_{i}$) of each component at a given composition and at that iteration temperature ($x_{i}$,T). The algorithm is halted once the left- and right-hand sides of Equation (2) match, within a given tolerance. The geometries of the compounds and corresponding σ-surfaces were optimized in the software package TURBOMOLE V7.4 2019 [48,49] using DFT with the BP-86 functional, the triple-ζ valence polarized basis set (TZVP), and the COSMO solvation model with infinite permittivity. Finally, the COSMOtherm [50] software package with the BP_TZVP_21 parametrization was used to perform all COSMO-RS-related calculations, including the aforementioned iterative algorithm. The σ-surfaces and geometries of the optimized molecules are depicted in Figure S7.

## 4. Conclusions

This work studied the feasibility of forming DES using menthol or thymol as HBD and flavonoids as HBA. It was shown that deep eutectic systems are formed by thymol and flavone or flavanone (lone HBA), showing strong negative deviations from ideality caused mainly by the formation of stronger hydrogen bonds. The observed experimental eutectic temperatures are below room temperature, making these mixtures very attractive for technical applications. On the other hand, the menthol eutectic systems showed ideal behavior, or positive deviations from ideality, as did the hesperetin-based systems. This difference in interactions may be used to selectively extract flavonoids that behave as lone HBA over more complex chemical structures such as hesperetin.

COSMO-RS displayed a remarkable ability to quantitatively predict all solid-liquid phase diagrams measured in this work, showing that it is a reliable tool to screen type V DES precursors. Furthermore, its accuracy allowed for a more in-depth study of the interactions present in the systems studied, particularly on how the excess enthalpies (and their main contributors) changed with the molecular structure of the compounds and the temperature of the system. Finally, COSMO-RS was also used to propose an alternative route to formulate hesperetin-based type V DES, using lone HBA, to be explored in the future.

## Figures and Tables

**Table 1:** Compound description and their melting properties.

Compound	Source	CAS	Purity wt. % *^a^*	Water Content wt. %	*T*_m_/K		Δ_m_*H*/kJ·mol^−1^
					This Work *^b^*	Lit.	
(−)-Menthol	Acros Organics *^c^*	89-78-1	≥99.5	<0.01	314.2 ± 0.3	315.68 ± 0.22 *^d^*	12.89 ± 0.77 *^d^*
Thymol	TCI Chemicals *^e^*	89-83-8	≥99.0	<0.01	323.3 ± 0.3	323.50 ± 0.34 *^d^*	19.65 ± 0.42 *^d^*
Flavone	Acros Organics *^c^*	525-82-6	≥99.0	<0.01	369.6 ± 0.3	369.89 ± 0.13 *^f^*	20.32 ± 0.14 *^f^*
Flavanone	Alfa Aesar *^g^*	487-26-3	≥98.0	<0.01	348.8 ± 0.3	349.49 ± 0.13 *^f^*	21.04 ± 0.19 *^f^*
(−)-Hesperetin	Cayman Chemical *^h^*	520-33-2	≥98.0	<0.282	504.8 ± 0.3	499.22 ± 1.00 *^i^*	35.9 ± 0.08 *^i^*

*^a^* Declared by the supplier; *^b^* Measured by glass capillary visual method; *^c^* Geel, Belgium; *^d^* Data from [33]; *^e^* Zwijndrecht, Belgium; *^f^* Data from [42]; *^g^* Kandel, Germany; *^h^* Ann Arbor, MI, USA; *^i^* Data from [43].

## Data Availability

Not applicable.

## Supplementary Materials

The following supporting information can be downloaded at: https://www.mdpi.com/article/10.3390/molecules27092649/s1, Tables S1–S6: Experimental solid-liquid equilibrium data for the systems including glass transitions (when applicable) and activity coefficients; Figure S1: DSC curves of multiple systems; Figure S2: Excess enthalpy predicted by COSMO-RS, at 298.2 K, of the studied systems; Figure S3: Excess enthalpy predicted by COSMO-RS, at 440.0 K, of hesperetin systems; Figure S4: Sigma-profile of flavone, flavanone, and hesperetin; Figure S5: Solid-liquid phase diagram data of multiple hesperetin systems extracted from diagrams reported by Chadha et al. (2017) [41]; Figure S6: Activity coefficient of multiple hesperetin systems using data reported by Chadha et al. (2017) [41]; Figure S7: Polarity surface (σ-surface) of thymol, menthol, and flavonoids.
