# Supplementary material for: Liquefying Flavonoids with Terpenoids through Deep Eutectic Solvent Formation
Source: Molecules. 2022 Apr 20;27(9):2649. doi: 10.3390/molecules27092649 (PMC9101798; doi:10.3390/molecules27092649)
Supplement: Supplementary file 1 [file molecules-27-02649-s001.zip › molecules-1652176 supplementary.pdf]

SUPPLEMENTARY INFORMATION

Liquefying Flavonoids with Terpenoids through Deep Eutectic Solvent Formation

Gabriel Teixeira[1], Dinis O. Abranches[1], Liliana P. Silva[1], Sérgio M. Vilas-Boas[1,2], Simão P. Pinho[2], Ana I. M. C. Lobo Ferreira[3], Luís M. N. B. F. Santos[3], Olga Ferreira[2,*], João A. P. Coutinho[1,*]

[1]CICECO − Aveiro Institute of Materials, Department of Chemistry, University of Aveiro, 3810-193 Aveiro, Portugal.

[2]Centro de Investigação de Montanha (CIMO), Instituto Politécnico de Bragança, Campus de Santa Apolónia, 5300-253 Bragança, Portugal.

[3]CIQUP, Institute of Molecular Sciences (IMS)- Departamento de Química e Bioquímica, Faculdade de Ciências da Universidade do Porto, Rua Campo Alegre, 4169-007 Porto, Portugal

Corresponding authors e-mail address: oferreira@ipb.pt (O.F.); jcoutinho@ua.pt (J.A.P.C.)

Number of Pages: 7

Number of Figures: 7

Number of Tables: 6

Tables

Table S.1 – Experimental solid-liquid equilibrium data (x_{thymol}, T_{eut}, T_m) for the system thymol/flavone, including glass transitions (T_g). The activity coefficients (γ) of thymol or flavone were obtained as per Eq. 2 using the melting properties reported in Table 1.

x_{thymol}	T_m (K)	T_{eut} (K)	T_g (K)	$\gamma_{flavone}$	x_{thymol}	T_m (K)	T_g (K)	γ_{thymol}
0.00 [a]	369.6	-	-	1.000	0.76 [b]	306	223	0.878
0.10 [a]	363	-	-	0.993	0.77 [c]	304	221	0.804
0.14 [b]	360	-	236	0.981	0.80 [c]	306	224	0.830
0.19 [a]	357	-	-	0.981	0.84 [b]	312	218	0.905
0.26 [c]	-	-	237	-	0.85 [c]	310	217	0.862
0.26 [b]	349	285	238	0.919	0.90 [a]	318	-	0.985
0.29 [b]	349	285	238	0.940	1.00 [a]	323.3	-	1.000
0.30 [a]	348	-	-	0.954				
0.30 [c]	338	-	243	0.778				
0.35 [c]	330	-	238	0.697				
0.40 [b]	318	286	240	0.571				
0.40 [c]	-	-	239	-				
0.41 [c]	-	-	240	-				
0.46 [c]	-	-	239	-				
0.50 [b]	-	-	239	-				
0.55 [b]	-	-	237	-				
0.61 [b]	-	-	233	-				
0.65 [b]	-	-	231	-				
0.70 [c]	-	-	226	-				
0.75 [c]	-	-	224	-				

[a] Visual method; [b] DSC 2; [c] DSC 1. Standard uncertainties, u, are $u_r(p)$ = 0.05, $u_r(x)$ = 0.01. For the temperatures measured by the visual method: $u(T)$ = 0.3 K for pure compounds and $u(T)$ = 1.9 K for mixtures. For experimental transition temperatures measured by DSC: $u(T)$ = 1 K for T_m, T_{eut} and T_g (including calibration uncertainty).

Table S.2 – Experimental solid-liquid equilibrium data (x_{thymol}, T_{eut}, T_m) for the system thymol/flavanone, including glass transitions (T_g). The activity coefficients (γ) of thymol or flavanone were obtained as per Eq. 2 using the melting properties reported in Table 1.

x_{thymol}	T_m (K)	T_{eut} (K)	T_g (K)	$\gamma_{flavanone}$	x_{thymol}	T_m (K)	T_g (K)	γ_{thymol}
0.00 [a]	348.8	-	-	1.000	0.49 [c]	-	234	-
0.10 [a]	345	-	-	1.025	0.51 [b]	-	230	-
0.20 [b]	332	-	226	0.877	0.63 [b]	-	223	-
0.25 [b]	331	-	228	0.904	0.70 [b]	299	223	0.794
0.27 [c]	330	268	227	0.914	0.74 [c]	305	223	0.874
0.31 [b]	324	-	235	0.840	0.75 [b]	304	222	0.841
0.41 [b]	313	-	-	0.747	0.80 [b]	306	219	0.830
0.41 [b]	-	-	231	-	0.90 [a]	316	-	0.941
0.46 [b]	310	-	232	0.745	0.90 [b]	314	213	0.879
					1.00 [a]	323.3	-	1.000

[a] Visual method; [b] DSC 1; [c] DSC 2. Standard uncertainties, u, are $u_r(p)$ = 0.05, $u_r(x)$ = 0.01. For the temperatures measured by the visual method: $u(T)$ = 0.3 K for pure compounds and $u(T)$ = 1.9 K for mixtures. For experimental transition temperatures measured by DSC: $u(T)$ = 1 K for T_m, T_{eut} and T_g (including calibration uncertainty).

Table S.3 – Experimental solid-liquid equilibrium data ($x_{menthol}$, T_m) for the system menthol/flavone obtained by the visual method. The activity coefficient (γ) of menthol or flavone were obtained as per Eq. 2 using the melting properties reported in Table 1.

$x_{menthol}$	T_m (K)	$\gamma_{flavone}$	$x_{menthol}$	T_m (K)	$\gamma_{menthol}$
0.00	369.6	1.000	0.90	308	1.018
0.10	365	1.030	0.95	311	1.008
0.20	362	1.081	1.00	314.2	1.000
0.30	357	1.139			
0.40	351	1.181			
0.50	345	1.256			
0.60	340	1.405			
0.70	330	1.513			
0.80	323	1.926			
0.85	317	2.228			

Standard uncertainties, *u*, are $u_r(p) = 0.05$, $u_r(x) = 0.01$. For the temperatures measured by the visual method: $u(T) = 0.3$ K for pure compounds and $u(T) = 1.9$ K for mixtures.

Table S.4 – Experimental solid-liquid equilibrium data ($x_{menthol}$, T_m) for the system menthol/flavanone obtained by the visual method. The activity coefficient (γ) of menthol or flavanone were obtained as per Eq. 2 using the melting properties reported in Table 1.

$x_{menthol}$	T_m (K)	$\gamma_{flavanone}$	$x_{menthol}$	T_m (K)	$\gamma_{menthol}$
0.00	348.8	1.000	0.95	312	1.012
0.10	344	1.017	1.00	314.2	1.000
0.21	341	1.073			
0.29	339	1.155			
0.40	336	1.264			
0.51	334	1.475			
0.60	330	1.678			
0.70	327	2.069			
0.80	324	2.909			
0.88	317	4.169			

Standard uncertainties, *u*, are $u_r(p) = 0.05$, $u_r(x) = 0.01$. For the temperatures measured by the visual method: $u(T) = 0.3$ K for pure compounds and $u(T) = 1.9$ K for mixtures.

Table S.5 – Experimental solid-liquid equilibrium data (x_{thymol},T_m) for the system thymol/hesperetin obtained by the visual method. The activity coefficients (γ) of thymol were obtained as per Eq. 2 using the melting properties reported in Table 1.

x_{thymol}	T_m (K)	$\gamma_{hesperetin}$
0.00	504.8	1.000
0.25	498	1.179
0.29	496	1.206
0.40	490	1.276
0.50	487	1.459
0.60	481	1.668
0.70	475	1.957
0.80	468	2.535
0.90	450	3.474
0.95	436	5.157
0.97	421	5.788
1.00	323.3	-

Standard uncertainties, *u*, are $u_r(p) = 0.05$, $u_r(x) = 0.01$. For the temperatures measured by the visual method: $u(T) = 0.3$ K for pure compounds and $u(T) = 1.9$ K for mixtures.

Table S.6 – Experimental solid-liquid equilibrium data ($x_{menthol}$, T_m) for the system menthol/hesperetin obtained by the visual method. The activity coefficient ($\boldsymbol{\gamma}$) of menthol were obtained as per Eq. 2 using the melting properties reported in Table 1.

$x_{menthol}$	T_m (K)	$\gamma_{hesperetin}$
0.00	504.8	1.000
0.75	476	2.327
0.81	465	2.524
0.86	459	3.029
0.89	442	2.831
0.95	417	3.213
1.00	314.2	-

Standard uncertainties, u, are $u_r(p) = 0.05$, $u_r(x) = 0.01$. For the temperatures measured by the visual method: $u(T) = 0.3$ K for pure compounds and $u(T) = 1.9$ K for mixtures.

Figures

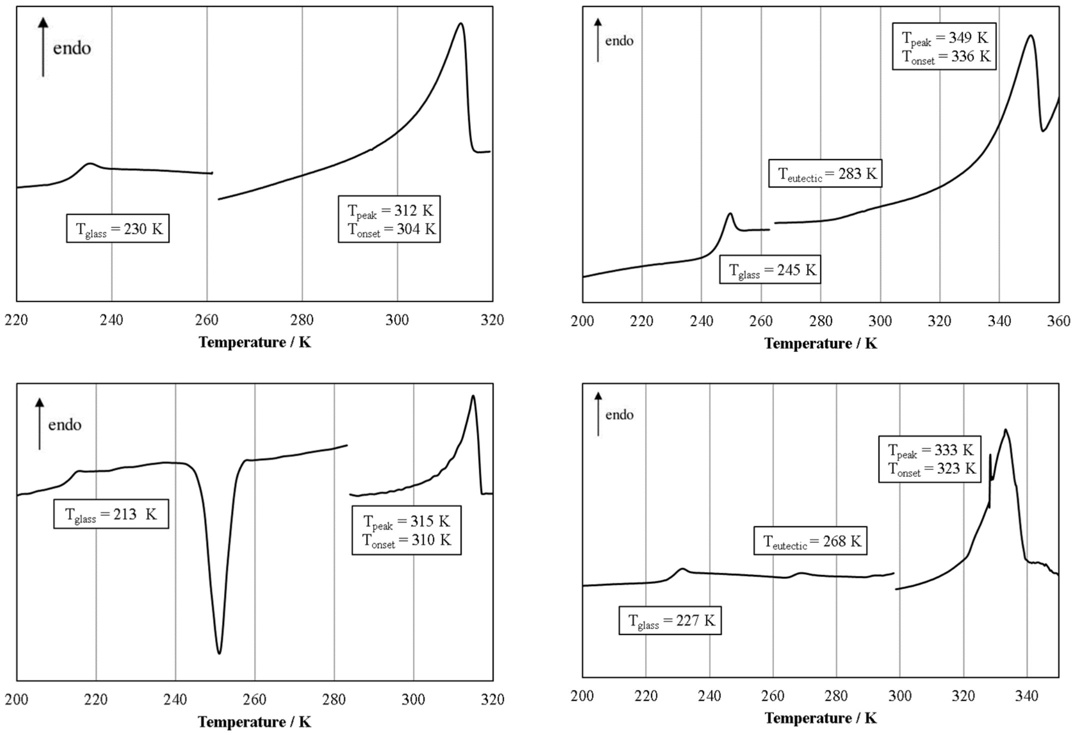

Figure S.1. DSC curves of the thymol/flavone system at x_{thymol} = 0.85 (top left / DSC 2) and x_{thymol} = 0.26 (top right / DSC 2) and the thymol/flavanone system at x_{thymol} = 0.90 (bottom left / DSC 1) and x_{thymol} = 0.27 (bottom right / DSC 2). The standard uncertainties for experimental transition temperatures obtained through DSC measurements were estimated to be 1 K for T_m , T_{eut} and T_g (including calibration uncertainty). The DSC curves are shifted for clarity.

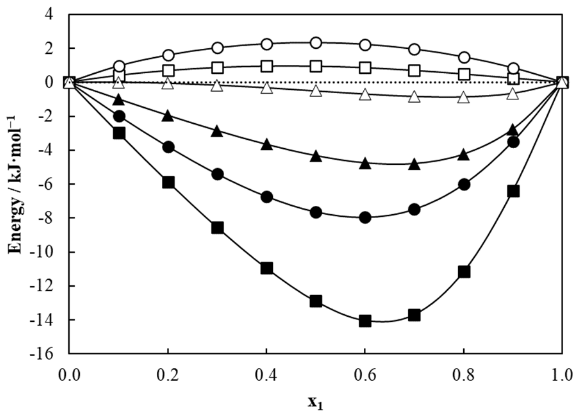

Figure S.2. Excess enthalpy predicted by COSMO-RS, at 298.2 K, of the systems with thymol (black symbols) and menthol (white symbols), and flavone (□), flavanone (○) and hesperetin (△). The dotted line represents the ideal behaviour.

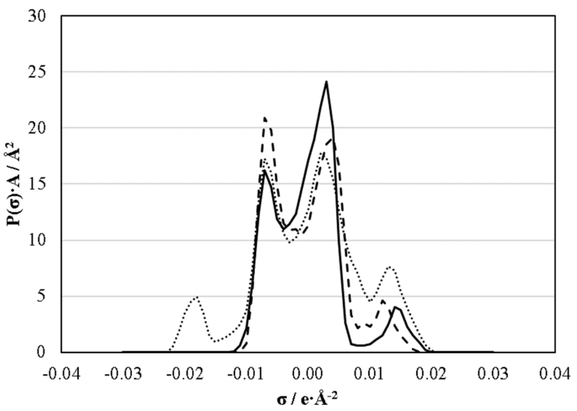

Figure S.3. Sigma-profile of flavone (—), flavanone (— —) and hesperetin (•••).

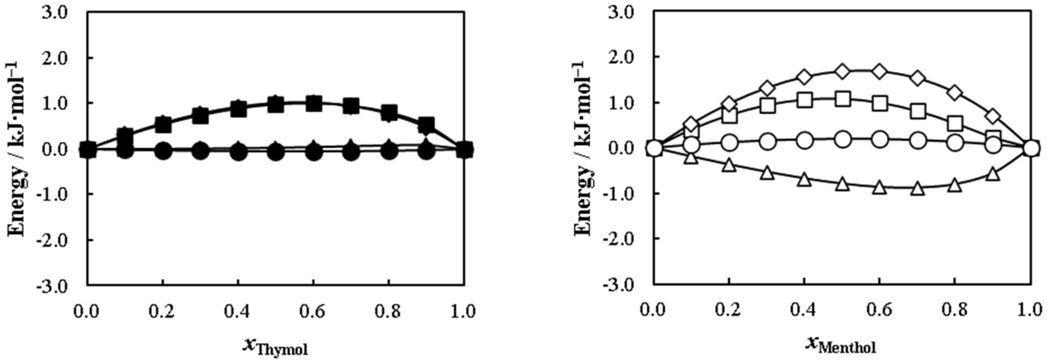

Figure S.4. Excess enthalpy predicted by COSMO-RS, at 440.0 K, of thymol/hesperetin (left) and menthol/hesperetin (right) mixtures. The symbols represent: total excess enthalpy (□), misfit contribution (◇), hydrogen bond contribution (△) and van der Waals contribution (○).

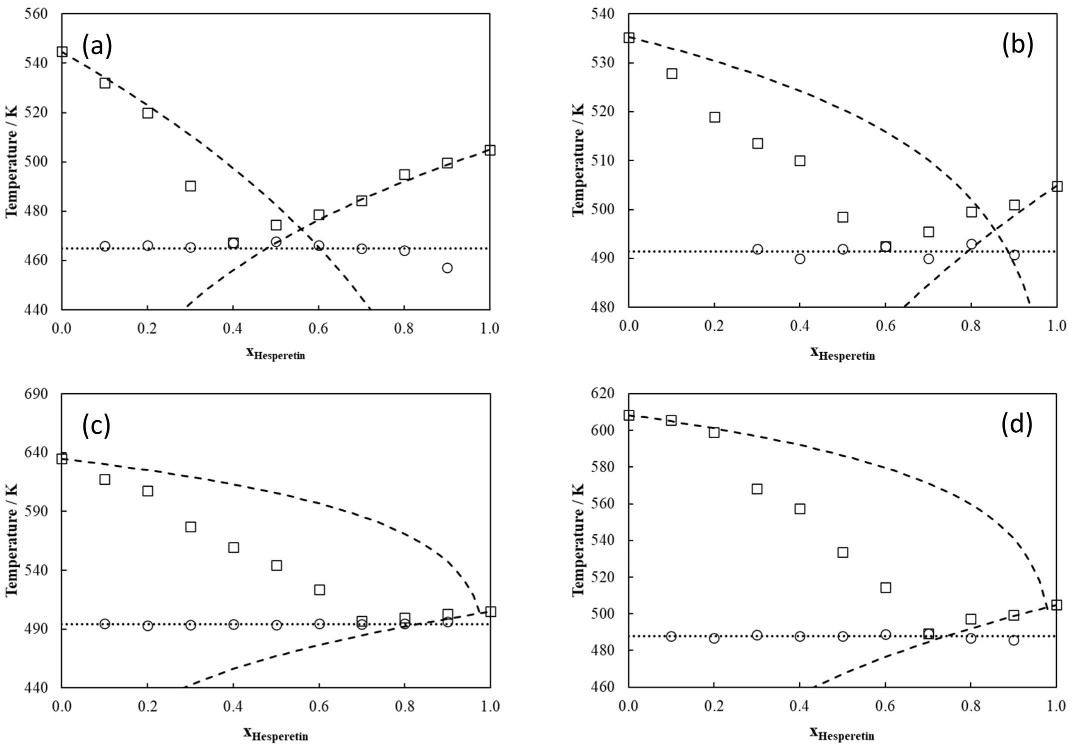

Figure S.5. Solid-liquid phase diagram data of hesperetin/theophylline (a), hesperetin/gallic acid (b), hesperetin/adenine (c), hesperetin/theobromine (d), extracted from diagrams reported by Chadha et al. (2017) [40]. Symbols represent literature data of melting temperature (□) and eutectic temperature (○). Lines represent the ideal melting curve of the system (— —) and eutectic temperature line (•••). Melting temperature and enthalpy used for the ideal curve calculation were 544.69 K and 24.38 kJ/mol for theophylline [40]; 535.25 K and 108.49 kJ/mol for gallic acid [40]; 634.94 K and 75.84 kJ/mol for adenine [40]; 608.37 K and 93.52 kJ/mol for theobromine [40]; and 504.86 K [40] and 35.90 kJ/mol [42] for hesperetin.

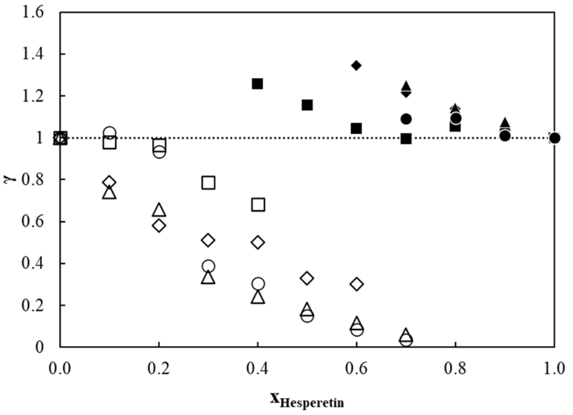

Figure S.6. Activity coefficient of hesperetin/theophylline (□), hesperetin/gallic acid (◇), hesperetin/adenine (△) and hesperetin/theobromine (○), using data reported by Chadha et al. (2017) [40]. The black symbols represent the activity coefficients of hesperetin and the white symbols, the activity coefficients of the other compound. Line represents the ideal behaviour (•••). Melting temperature and enthalpy used for the calculation are the same of Figure S.5.

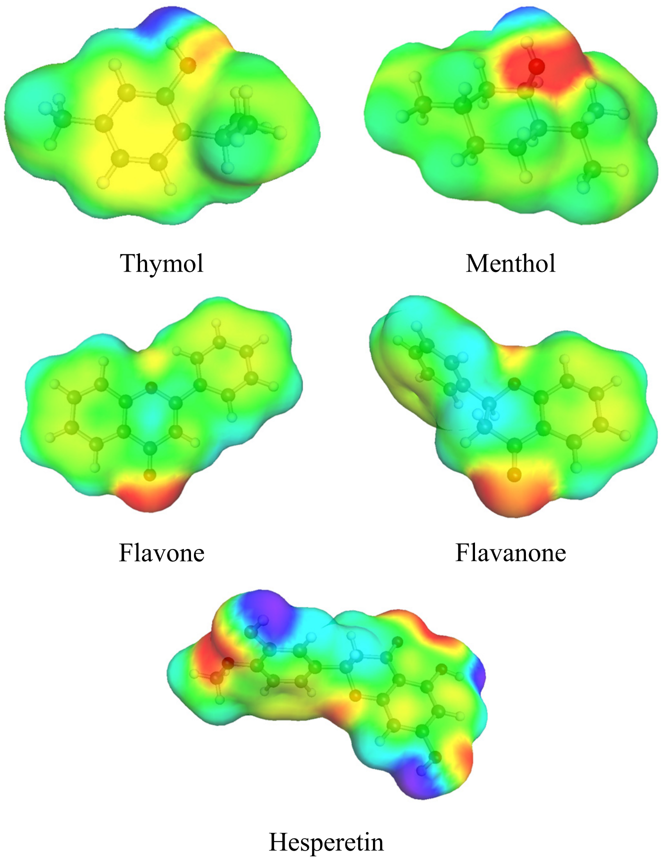

Figure S.7. Polarity surface (σ-surface) of thymol, menthol, and flavonoids.
